# Effect of differentiation on microRNA expression in bovine skeletal muscle satellite cells by deep sequencing

**DOI:** 10.1186/s11658-016-0009-x

**Published:** 2016-07-28

**Authors:** Wei Wei Zhang, Xiao Feng Sun, Hui Li Tong, Ya Hui Wang, Shu Feng Li, Yun Qin Yan, Guang Peng Li

**Affiliations:** 1 The Laboratory of Cell and Development, Northeast Agricultural University, Mucai Street 59, Xiangfang District, Harbin, 150030 Heilongjiang China; 2 College of Life Sciences and Agriculture & Forestry, Qiqihar University, Qiqihar, Heilongjiang 161006 China; 3 The Key Laboratory of Mammal Reproductive Biology and Biotechnology Ministry of Education, Inner Mongolia University, Hohhot, 010021 China

**Keywords:** Bovine, Skeletal muscle-derived satellite cells, Deep sequencing, miRNA, Differentiation, Proliferation, Differentially expressed miRNA, Target prediction, Gene Ontology, KEGG analysis

## Abstract

**Background:**

The differentiation of skeletal muscle-derived satellite cells (MDSCs) is important in controlling muscle growth, improving livestock muscle quality, and healing of muscle-related disease. MicroRNAs (miRNAs) are a class of gene expression regulatory factors, which play critical roles in the regulation of muscle cell differentiation. This study aimed to compare the expression profile of miRNAs in MDSC differentiation, and to investigate the miRNAs which are involved in MDSC differentiation.

**Method:**

Total RNA was extracted from MDSCs at three different stages of differentiation (MDSC-P, MDSC-D1 and MDSC-D3, representing 0, 1 and 3 days after differentiation, respectively), and used to construct small RNA libraries for RNA sequencing (RNA-seq).

**Results:**

The results showed that in total 617 miRNAs, including 53 novel miRNA candidates, were identified. There were 9 up-expressed, 165 down-expressed, and 15 up-expressed, 145 down-expressed in MDSC-D1 and MDSC-D3, respectively, compared to those in MDSC-P. Also, 17 up-expressed, 55 down-expressed miRNAs were observed in MDSC-D3 compared to those in MDSC-D1. All known miRNAs belong to 237 miRNA gene families. Furthermore, we observed some sequence variants and base edits of the miRNAs. GO and KEGG pathway analysis showed that the majority of target genes regulated by miRNAs were involved in cellular metabolism, pathways in cancer, actin cytoskeleton regulation and the MAPK signaling pathway. Regarding the 53 novel miRNAs, there were 7 up-expressed, 31 down-expressed, and 8 up-expressed, 26 down-expressed in MDSC-D1 and MDSC-D3, respectively, compared to those in MDSC-P. The expression levels of 12 selected miRNA genes detected by RT-qPCR were consistent with those generated by deep sequencing.

**Conclusions:**

This study confirmed the authenticity of 564 known miRNAs and identified 53 novel miRNAs which were involved in MDSC differentiation. The identification of novel miRNAs has significantly expanded the repertoire of bovine miRNAs and could contribute to advances in understanding muscle development in cattle.

**Electronic supplementary material:**

The online version of this article (doi:10.1186/s11658-016-0009-x) contains supplementary material, which is available to authorized users.

## Background

In livestock, all muscle fibers are formed during the prenatal stage. Postnatal growth and regenerative ability of adult skeletal muscles are dependent on adult muscle stem cells known as satellite cells, which reside beneath the basal lamina of the mature fibers [1, 2]. In the process of MDSC differentiation, progenitor cells first proliferate, then exit from the cell cycle, and undergo differentiation, alignment, and fusion to form multinucleated myotubes [3–5]. These molecular events are orchestrated by myogenic regulatory factors and miRNAs. The miRNAs are a family of noncoding small RNAs, approximately 22 nucleotides in length. They can repress the translation and accelerate the decay of mRNAs through pairing of their seed sequences with the 3′ UTRs of target genes [6]. The miRNAs have been shown to play critical roles in skeletal muscle development and in regulation of muscle cell proliferation and differentiation [7]. For example, miR-1 and miR-206 could promote the differentiation of myoblasts, whereas miR-133 could promote cell proliferation [8–10]. Additionally, miR-27 could modulate the entry of cells into the myogenic differentiation program [11].

Recently, Sun et al. identified conserved and novel miRNAs from the Chinese Qinchuan bovine longissimus thoracis by high-throughput sequencing [12]. There have been few studies on their involvement in the regulation of MDSC differentiation. The aim of this study was to investigate the miRNA expression profiles in MDSCs during the differentiation process by using RNA-seq. Elucidation of the expression patterns of different miRNA among different differentiation stages would contribute to our understanding of the roles of miRNAs in gene expression regulatory networks during the differentiation of MDSCs.

## Methods

### Ethics statement

Sample collection from animals was approved by the Animal Care Commission of the Northeast Agricultural University and Heilongjiang, P.R. China. Skeletal muscle tissues of the newborn Chinese Simmental calves were collected from Shuangcheng abattoir, a local slaughterhouse in Heilongjiang, Peoples Republic of China.

### MDSC culture and differentiation

MDSCs were isolated from hindlimb muscles of three newborn Chinese Simmental calves according to the method described by Lee et al. [13] and Tong et al. [14]. The MDSCs were cultured in growth medium (GM). For MDSC differentiation, cells were seeded on plates at a density of 4–10^5^ cells/60-mm dishes, and allowed to adhere for 24 h in GM. Subsequently, the cells were switched to differentiation medium (DM) containing 2 % horse serum (Gibco), 100 U/mL penicillin, and 100 μg/mL streptomycin in Dulbecco’s modified Eagle’s medium. The MDSCs were collected after switching to DM for 0 days (MDSC-P), 1 day (MDSC-D1) and 3 days (MDSC-D3). Each differentiation stage had three replicates which came from the three cattle mentioned above. The differentiation of MDSCs was evaluated by the immunolocalization of MHC and desmin genes according to the method described by Tong et al. [14], and the relative mRNA expression levels of PAX3, MYOD, MYF5, MYF6, MYOG and MHC genes which were involved in the cell differentiation. All reactions were performed in triplicate and the relative expression level of mRNA was normalized to the expression of the *β-actin* gene. The quantification of each mRNA relative to the *β-actin* gene was calculated by the formula: N = 2^−ΔΔCt^.

### RNA extraction and high-throughput sequencing

Total RNA was extracted from MDSC samples using TRI reagent (Invitrogen, Carlsbad, CA) following the manufacturer’s instructions. The quantity of total RNA was measured using an Agilent 2100 Bioanalyzer with Agilent RNA 6000 nano Reagents Port 1 kit (Agilent Technologies, Santa Clara, CA, USA). Optical density values at 260/280 were consistently above 1.9. The samples with intact, distinct ribosomal peaks were chosen for further analysis. The RNAs from three replicates were pooled as one RNA sample at each differentiation stage of MDSCs. Subsequently, the RNAs with low molecular weight were separated by 15 % PAGE, and the RNAs with molecules in the range of 18–30 nt were enriched and ligated with proprietary adapters to the 5' and 3' termini. A reverse transcription reaction followed by low-cycle PCR was performed to obtain sufficient product for high-throughput sequencing in Beijing Genomics Institute, China.

### Small RNA sequence analysis

After removing the 3′ adaptor sequence and removal of redundancy and reads smaller than 18 nt, the clean reads were screened against and mapped to the latest bovine genome assembly (ftp://hgdownload.cse.ucsc.edu/goldenPath/bosTau7/bigZips/bosTau7.fa.gz) using the program SOAP [15]. To identify sequences originating from protein-coding genes, repeats, rRNA, tRNA, snRNA, and snoRNA, the bovine mRNA (ftp://hgdownload.cse.ucsc.edu/goldenPath/bosTau7/database/refGene.txt.gz) and CDS, Repeat Masker (ftp://hgdownload.cse.ucsc.edu/goldenPath/bosTau6/bigZips/bosTau7.fa.out.gz) and Sanger Rfam data (version 10.1) were used. The remaining reads were searched against the Sanger miRBase (version 21.0) database to identify conserved miRNAs. Only those small RNAs whose mature and precursor sequences perfectly matched known bovine miRNAs in miRBase were considered to be conserved miRNAs. To discover potential novel miRNA precursor sequences, unique sequences that had more than 10 hits to the genome or matched known noncoding RNAs were removed. Subsequently, the flanking sequences (150 nt upstream and downstream) of each unique sequence were extracted for secondary structure analysis with Mfold (http://www.bioinfo.rpi.edu/applications/mfold) and then evaluated by Mireap (http://sourceforge.net/projects/mireap/). The criteria [16] were used to screen candidates for potential miRNAs or pre-miRNAs as follows: [1] pre-miRNA sequences could fold into an appropriate hairpin secondary structure that contained the ~22 nt mature miRNA sequence within one arm of the hairpin. [2] miRNA precursors with secondary structures had higher negative minimal free energies (MFEs) and minimal free energy indexes (MFEIs) than other different types of RNAs. [3] miRNA had an AU content of 30–70 %. [4] miRNA had less than six mismatches with the opposite miRNA* sequence in the other arm. [5] No loop or break in miRNA sequences was allowed. After prediction, the resulting potential miRNA loci were examined carefully based on the distribution and numbers of small RNAs in the entire precursor regions. Those sequences residing in the stem region of the stem-loop structure and ranging between 20 and 22 nt with free energy hybridization lower than −20 kcal/mol were considered [17].

To predict the target genes of miRNAs, we used the RNAhybrid software program for target prediction (ftp://hgdownload.cse.ucsc.edu/goldenPath/bosTau7/bigZips/refMrna.fa.gz). This program was based on the criteria suggested by Allen et al. [18] and Schwab et al. [19]. Finally, to reveal the functions of the putative target genes, Gene Ontology (GO) analysis was performed on the predicted target gene candidates of the novel miRNAs using three ontologies: molecular function, cellular components, and biological process. The functions of the putative target genes which were regulated by miRNAs were determined using KEGG pathway analysis.

### MicroRNA expression analysis

Expression levels of known miRNAs between two samples were compared to identify differentially expressed miRNAs. The expression of miRNA was shown in two samples by plotting the log_2_ ratio and making a scatter plot. The procedures were as follows. [1] Normalize the expression of miRNA in three samples (MDSC-P, MDSC-D1, and MDSC-D3) to obtain the expression of transcripts per million. Normalized expression (NE) = actual miRNA count/total count of clean reads. [2] Calculate the fold-change and P-value from the normalized expression. Then, generate the log_2_ ratio plot and scatter plot. Fold-change formula: Fold-change formula: fold-change = log_2_ (MDSC-D1 or MDSC-D3/MDSC-P). *P*-value formula: $p\left(x/b\right)={\left(\frac{N_2}{N_1}\right)}^y{\frac{\left(x+y\right)!}{x!y!{\left(1+\frac{N_2}{N_1}\right)}^{\left(x+y+1\right)}}}_{D\left(y\ge {y}_{\max }/x\right)={\displaystyle \sum_{y\ge {y}_{\max}}^{\infty }p\left(y/x\right)}}^{C\left(y\le {y}_{\min }/x\right)={\displaystyle \sum_{y=0}^{y\le {y}_{\min }}p\left(y/x\right)}}$, where *x* and *y* represent normalized expression levels, and *N*_1_ and *N*_2_ represent total counts of clean reads of a given miRNA in small RNA libraries of the MDSC-P, MDSC-D1, and MDSC-D3 stages, respectively.

### RT-qPCR

Twelve miRNAs were randomly chosen for verification of RNA-seq results by RT-qPCR [20]. The same RNA samples used for RNA-seq library preparation and sequencing were used for the RT-qPCR. Total RNA (1 μg) was converted to cDNA with an RT primer mixture (250 nM) using the BioTake Super RT Kit (BioTake, Beijing, China). The cDNA was then used for qPCR of miRNA using the miRNA-specific primer and the universal primer. The bovine 18S ribosomal RNA (18S) gene was used as a reference gene. The primers for miRNAs and the reference gene are listed in Additional file 1. RT-qPCR reaction was performed on an ABI7300 Real Time Detection System. The cycle conditions were as follows: 95 °C for 2 min, followed by 40 cycles of 95 °C for 15 s, and 60 °C for 30 s. The miRNA expression was normalized to the expression of the 18S rRNA gene and calculated using the 2^−ΔΔCt^ method. All reactions were performed in triplicate, and the RT-qPCR results among different treatment groups were statistically tested using one-way ANOVA by Tukey’s test at *p* = 0.05.

## Results

### Evaluation of MDSC differentiation

**Fig. 1:**
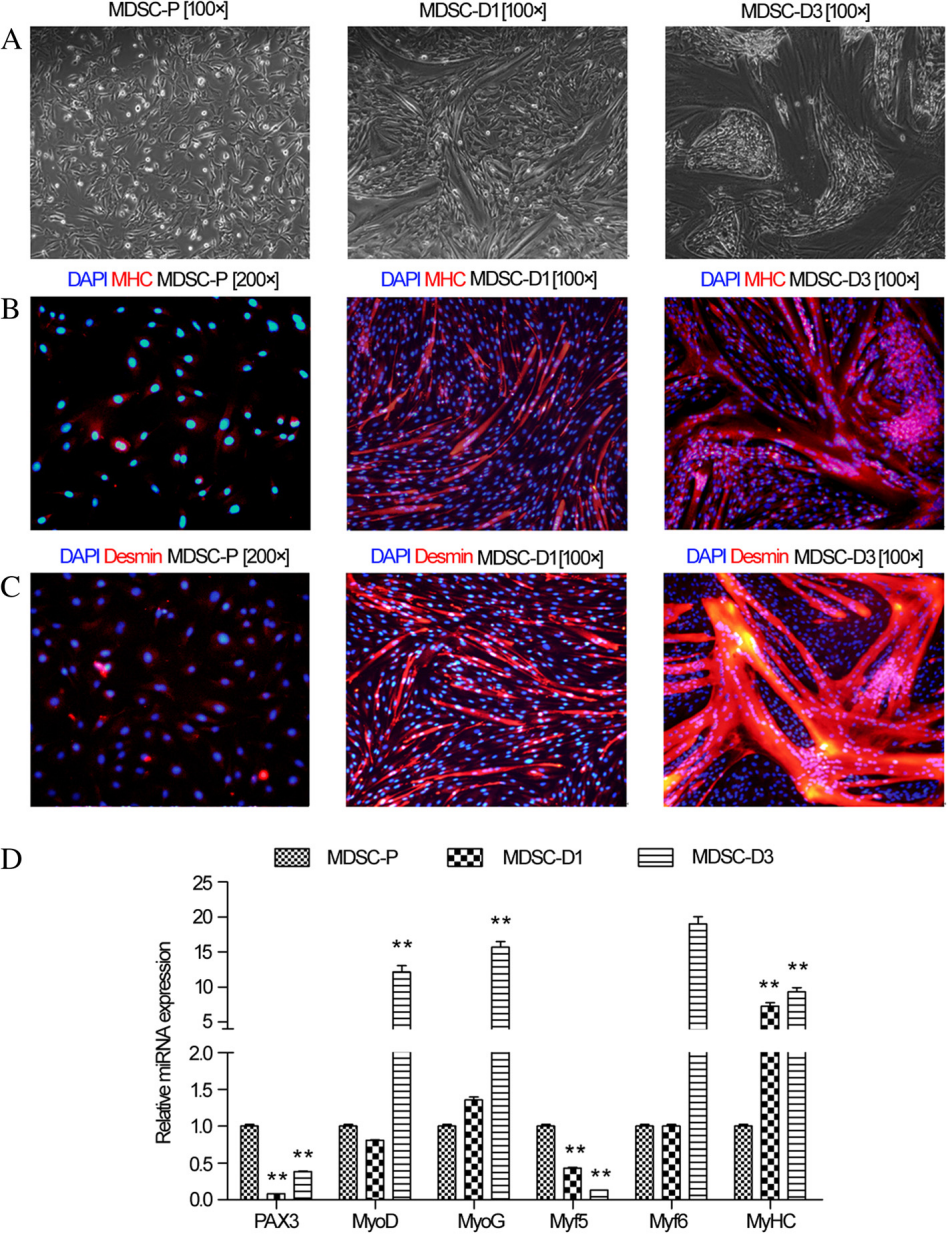
Morphology and immunofluorescence characterization of MDSCs. **a** Morphology of MDSCs during differentiation for 0, 1, and 3 days (MDSC-P, MDSC-D1, and MDSC-D3, respectively); **b** Immunofluorescence detection of MHC in MDSCs during differentiation at MDSC-P, MDSC-D1 and MDSC-D3; **c** Immunofluorescence detection of desmin in MDSCs during differentiation at MDSC-P, MDSC-D1 and MDSC-D3. **d** Expression of MRF during MDSC differentiation. The RT-qPCR analysis of PAX3, MYOD, MYOG, MYF5, and MYF6 genes in MDSC-D1 and MDSC-D3 compared to MDSC-P. Error bar indicates standard error of mean of triplicate samples. *p < 0.05,**p < 0.01

Cell differentiation was evaluated by morphology, MHC and desmin immunolocalization, as well as MRF gene expression. Bovine MDSCs were derived from the primary culture of muscle tissue and fused myotubes were generated by culturing MDSCs in DM for 1 and 3 days (Fig. 1a). In MDSC-P, the MHC and desmin genes were not expressed, but they were in MDSC-D1 and MDSC-D3 (Fig. 1b, c). The analysis of MRF gene expression showed the PAX3 and MYF5 genes were down-regulated, while MYOG, MYF6, and MHC genes were up-regulated in MDSC-D1 and MDSC-D3 compared to those in MDSC-P (Fig. 1d). These results indicated that the MDSCs were in the differentiation process in MDSC-D1 and MDSC-D3 but not MDSC-P, in which the MDSCs were only in proliferation.

### Cell collection and high-throughput sequencing of small RNAs

**Fig. 2:**
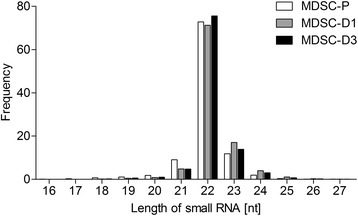
Length distributions of small RNAs in the three RNA libraries. White columns represent length distributions of small RNAs in the MDSC-P library; gray columns represent length distributions of small RNAs in the MDSC-D1 library; black columns represent length distributions of small RNAs in the MDSC-D3 library

To identify small RNAs in MDSCs during differentiation, total RNAs from MDSCs at different differentiation stages were used to construct small RNA libraries. We obtained 5,871,055 clean reads from the MDSC-P library, 5,922,188 from the MDSC-D1 library and 5,935,963 from the MDSC-D3 library after deleting some contaminant reads (Table 1). Length distribution analysis showed that most reads ranged from 21 to 23 nt. The percentage of 22-nt reads in total reads was 72.76, 71.26, and 75.58 % in the MDSC-P, MDSC-D1, and MDSC-D3 library, respectively (Fig. 2). The reads for the three libraries (5,188,810, 5,041,921 and 5,236,492, respectively) were perfectly matched to the bovine genome (Table 2).

**Table 1:** Summary of small RNA sequencing data

Type	MDSC-P		MDSC-D1		MDSC-D3	
	Count	%	Count	%	Count	%
total_reads	6000000		6000000		6000000	
high_quality	5984373	100 %	5984335	100 %	5984569	100 %
3'adaptor_null	2916	0.05 %	2967	0.05 %	2901	0.05 %
insert_null	3115	0.05 %	1252	0.02 %	1408	0.02 %
5'adaptor_contaminants	86464	1.44 %	54566	0.91 %	39651	0.66 %
smaller_than_18nt	20713	0.35 %	3297	0.06 %	4615	0.08 %
polyA	110	0.00 %	65	0.00 %	31	0.00 %
clean_reads	5871055	98.11 %	5922188	98.96 %	5935963	99.19 %

Note: total_reads: total sequenced reads, which is required to be greater than 5 M in general; high_quality: number of high quality reads with no N, no more than 4 bases whose quality score was lower than 10 and no more than 6 bases whose quality score was lower than 13; 3'adaptor_null: number of reads with no 3'adaptor; insert_null: number of reads with no insertion; 5'adaptor_contaminants: number of 5'contaminants; smaller_than_18nt: number of reads less than 18 nt generally. Small RNA tags were between 18 and 30 nt long, so too short tags should be removed from data for further analysis; polyA: number of reads with polyA; clean_reads: number of clean reads after adaptors and contaminants were removed, which were used in the following analysis

**Table 2:** Distribution of the genome-mapped sequence reads in small RNA libraries

Locus class	MDSC-P		MDSC-D1		MDSC-D3	
	Unique sRNAs	Total sRNAs	Unique sRNAs	Total sRNAs	Unique sRNAs	Total sRNAs
Total	106806 (100 %)	5871055 (100 %)	68492 (100 %)	5922188 (100 %)	66603 (100 %)	5935963 (100 %)
miRNA	3056 (2.86 %)	4844028 (82.51 %)	2328 (3.40 %)	4850321 (81.90 %)	2483 (3.73 %)	5051419 (85.10 %)
exon_antisense	473 (0.44 %)	1428 (0.02 %)	164 (0.24 %)	410 (0.01 %)	141 (0.21 %)	467 (0.01 %)
exon_sense	5111 (4.79 %)	7313 (0.12 %)	3167 (4.62 %)	4056 (0.07 %)	2892 (4.34 %)	3564 (0.06 %)
intron_antisense	2759 (2.58 %)	4632 (0.08 %)	899 (1.31 %)	1355 (0.02 %)	874 (1.31 %)	1201 (0.02 %)
intron_sense	4057 (3.80 %)	7573 (0.13 %)	1823 (2.66 %)	2844 (0.05 %)	1337 (2.01 %)	2175 (0.04 %)
rRNA	9082 (8.50 %)	64109 (1.09 %)	9408 (13.74 %)	79061 (1.33 %)	9454 (14.19 %)	108407 (1.83 %)
repeat	7322 (6.86 %)	15735 (0.27 %)	2490 (3.64 %)	4508 (0.08 %)	2208 (3.32 %)	4167 (0.07 %)
scRNA	104 (0.10 %)	1025 (0.02 %)	120 (0.18 %)	2064 (0.03 %)	134 (0.20 %)	2295 (0.04 %)
snRNA	591 (0.55 %)	2554 (0.04 %)	461 (0.67 %)	2024 (0.03 %)	332 (0.50 %)	1454 (0.02 %)
snoRNA	583 (0.55 %)	1666 (0.03 %)	568 (0.83 %)	1496 (0.03 %)	487 (0.73 %)	1374 (0.02 %)
srpRNA	66 (0.06 %)	134 (0.00 %)	100 (0.15 %)	175 (0.00 %)	85 (0.13 %)	312 (0.01 %)
tRNA	5712 (5.35 %)	48058 (0.82 %)	3893 (5.68 %)	29959 (0.51 %)	4428 (6.65 %)	35856 (0.60 %)
unann	67890 (63.56 %)	872800 (14.87 %)	43071 (62.88 %)	943915 (15.94 %)	41748 (62.68 %)	723272 (12.18 %)

### Identification of conserved bovine miRNAs

To identify conserved miRNAs in MDSCs during differentiation, the small RNAs with a length of 18–23 nucleotides were Blastn searched against miRBase 21.0 (miRBase release version V21.0, July 3, 2014). The miRNA candidates were then clustered into 439, 394, and 392 categories corresponding to 455, 412, and 410 independent genomic loci in the three libraries according to sequence similarity (Table 3), of which 322 miRNAs overlapped in three libraries (Additional file 2).

**Table 3:** Summary of known miRNA in RNA libraries

	miR	miR*	miR-5p	miR-3p	pre-miRs	Unique matched to pre-miRs	Read matched to pre-miRs
Known miRs	598	0	79	78	766	-	-
MDSC-P	339	0	51	49	455	3081	4844156
MDSC-D1	297	0	49	48	412	2352	4850401
MDSC-D3	297	0	46	49	410	2501	5051499

Note: * means minor miR sequences

**Fig. 3:**
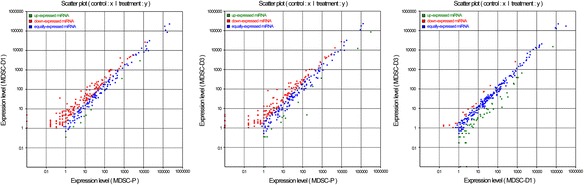
Differential expression of conserved miRNAs during differentiation of MDSCs. Compare the known miRNA expression between different differentiation stages to find out the differentially expressed miRNA. Each point in the figure represents a miRNA. The X axis and Y axis show the expression level of miRNAs in two libraries. Green points represent miRNAs with ratio > 2; Blue points represent miRNAs with ½ < ratio ≤2; Red points represent miRNAs with ratio ≤ 1/2. Ratio = Normalized expression in treatment/Normalized expression in control

The expression of known miRNAs was demonstrated by generating log_2_ ratio plots and scatter plots (Fig. 3). The expression profiles between the different libraries are shown in Additional file 3. The results showed that 296 miRNAs comprised 9 up-expressed (Table 4), 165 down-expressed, and 122 equally expressed miRNAs in MDSC-D1 compared to those in MDSC-P; 304 miRNAs comprised 15 up-expressed (Table 4), 145 down-expressed, and 144 equally expressed miRNAs in MDSC-D3 compared to those in MDSC-P; 273 miRNAs comprised 17 up-expressed, 55 down-expressed, and 201 equally expressed miRNAs in MDSC-D3 compared to those in MDSC-D1. Additional file 4: Table S1 lists the 10 most abundant miRNAs in MDSC-P, MDSC-D1, and MDSC-D3. The miRNA with the greatest count in MDSC-D3 was miR-206, a major miRNA in skeletal muscle development, with an average normalized read count of >1 million. The ubiquitously expressed let-7 members, let-7a-5p, let-7f, and let-7b, followed, and miR-1 had the fifth greatest count. The miRNA expression patterns during MDSC differentiation were clustered using hierarchical cluster analysis (Additional file 5: Figure S1). For example, compared with the proliferation stages (MDSC-P), the expression levels of miR-2443, miR-423-5p, miR-181a, miR-10a, and miR-206 were higher in MDSC-D1, and the pattern was the same as that of miR-139, miR-1, miR-95, miR-206, and miR-133a in MDSC-D3.

**Table 4:** miRNAs highly expressed in MDSC-D1 and MDSC-D3 compared with MDSC-P

Category	miRNA	MDSC-P	MDSC-D1	MDSC-D3	log2 Ratio (Fold Change)
MDSC-D1/MDSC-P	bta-miR-99a-3p	11	35		1.66
	bta-miR-2396	2	6		1.57
	bta-miR-139	27	67		1.29
	bta-miR-2443	51	121		1.23
	bta-miR-423-5p	16887	38687		1.18
	bta-miR-181a	2105	4694		1.14
	bta-miR-10a	24	53		1.13
	bta-miR-2331-5p	7	15		1.08
MDSC-D3/MDSC-P	bta-miR-2904	0		7	6.88
	bta-miR-139	27		990	5.18
	bta-miR-1	69315		408668	2.54
	bta-miR-2411-5p	2		9	2.15
	bta-miR-95	18		78	2.09
	bta-miR-99a-3p	11		46	2.04
	bta-miR-206	493002		1890636	1.92
	bta-miR-2396	2		7	1.79
	bta-miR-421	119		361	1.58
	bta-miR-500	5		15	1.56
	bta-miR-874	2		6	1.56
	bta-miR-10a	24		64	1.39
	bta-miR-503-5p	2066		5503	1.39
	bta-miR-362-5p	20		53	1.39
	bta-miR-133a	774		1994	1.34

Note: Fold Change = MDSC-D1/ MDSC-P; Fold Change = MDSC-D3/ MDSC-P

Nucleotide bias analysis at each position showed that GC content was high at the 2nd, 11th, and 15th positions with values of 94.34, 92.53, and 95.16 %, respectively, but not at the 1st, 6th, 9th, 10th, 13th, 14th, 16th, 22nd, and 24th positions with values of 0.70, 6.69, 4.94, 5.91, 4.69, 3.69, 3.40, 7.17, and 0.18 %, respectively, in the MDSC-P library. A similar result was obtained in the MDSC-D1 and MDSC-D3 libraries. In all libraries, nucleotides A + U were distributed mainly in the remaining positions with the exception of the 4th, 5th, 8th, 12th, 20th, and 23rd positions (Additional file 6: Figure S2). The phenomenon of nucleotide bias might be related to the mechanisms of miRNA, such as binding with targets for gene regulation. The 1st, 9th, and terminal positions were enriched with U and the 1st and 9th positions were the limits of the “seed region” of a miRNA which was responsible for targeting mRNAs for gene regulation [21]. A similar result was obtained in MDSC-P miRNAs at the 1st, 9th, and end positions, but there was only 44.35 % U at the end position in MDSC-D1 and 58.21 % at the 9th position in the MDSC-D3 library. The differences observed between our study and previous studies might have been due to different experimental approaches or differences in samples [12].

Positions 2–8 of a mature miRNA are called the seed region, which are highly conserved. The target of a miRNA might differ with changes of nucleotides in this region. In our study, miRNAs that might have a base edit could be detected by aligning unannotated sRNA tags with mature miRNAs from miRBase21, allowing one mismatch at a given position. The results showed that the mismatches occurred in all three libraries with the percentage of 22.65, 22.45, and 23.53 %, respectively. The mismatches could be caused by post-transcriptional modification, and/or RT-PCR, and sequencing errors (Additional file 7).

In our study, further analysis identified a total of 439, 394, and 392 conserved miRNAs that belonged to 237 miRNA families in the three libraries mentioned above. The largest miRNA family identified was miR-2284, which consisted of 63 members, and miR-154, let-7, and miR-181/30 possessed 18, 12, and 6 members, respectively; other miRNA families, such as miR-122, miR-1249, miR-140, and miR-486, had only one member, whereas miR-1940, miR-2286, miR-3431, and miR-574 did not belong to any gene family (Additional file 8).

### Identification of novel bovine miRNAs

The characteristic hairpin structure of a miRNA precursor could be used to predict novel miRNAs. The prediction software Mireap was developed to predict novel miRNAs by exploring the secondary structure, the Dicer cleavage site, and the minimum free energy of the unannotated small RNA reads that could be mapped to a genome. Based on HiSeq deep sequencing, 53 novel bovine miRNAs were identified in bovine MDSCs, which corresponded to 145 genomic loci. Forty-three novel miRNAs were in the MDSC-P library, 17 were in the MDSC-D1, and 19 were in the MDSC-D3 library, and 26 of the miRNAs overlapped in three libraries (Additional file 9). The read numbers from HiSeq deep sequencing are often regarded as a reliable quantification of miRNA expression; the read numbers of miRNAs in the HiSeq deep sequencing analysis are shown in Additional file 10.

The expression of novel miRNAs in MDSC-P, MDSC-D1, and MDSC-D3 was demonstrated by generating log_2_ ratio plots and scatter plots (Additional file 11: Figure S3). The results showed that 42 miRNAs comprised 7 up-expressed, 31 down-expressed, and 4 equally expressed miRNAs in MDSC-D1 compared to those in MDSC-P; 43 miRNAs comprised 8 up-expressed, 32 down-expressed, and 3 equally expressed miRNAs in MDSC-D3 compared to those in MDSC-P; and 24 miRNAs comprised 9 up-expressed, 9 down-expressed, and 6 equally expressed miRNAs in MDSC-D3 compared to those in MDSC-D1.

### qPCR analysis confirmed differential expression of selected miRNAs in MDSCs

**Fig. 4:**
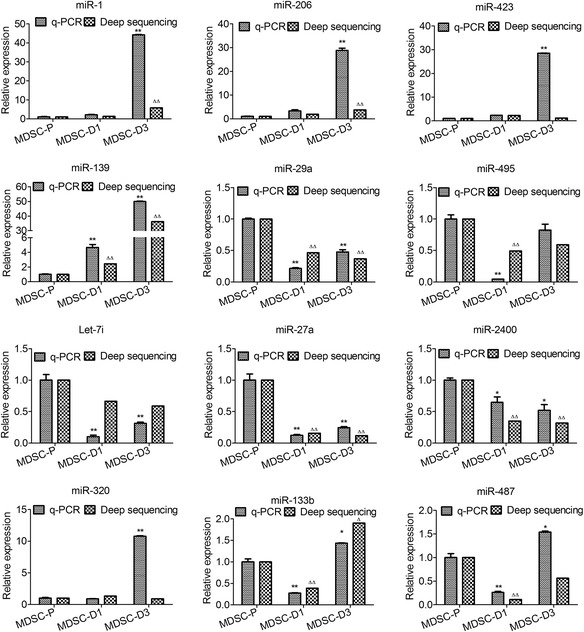
Expression of miRNAs during MDSC differentiation in bovine detected by RT-qPCR. Note: MDSCs during differentiation at 0, 1, and 3 days (MDSC-P, MDSC-D1, and MDSC-D3, respectively); Error bar indicates standard error of mean of triplicate samples. (* *p* < 0.05,** *p* < 0.01, compared with MDSC-P by q-PCR. ^∆^ *p* < 0.05, ^∆∆^ *p* < 0.01, compared with MDSC-P by deep sequencing)

To confirm the RNA-seq results, the expression of 12 miRNAs was quantified by stem-loop qPCR [22]. The results showed that miR-29a, miR-27a, and let-7i were highly expressed in MDSC-P; in contrast, miR-320, miR-1, and miR-206 were highly expressed in MDSC-D3. In addition, miR-206, miR-1, and miR-320 were up-regulated during MDSC differentiation; miR-495, miR-133b, and miR-487 were down-regulated in MDSC-D1 and upregulated in MDSC-D3. The results of the RT-qPCR analysis were consistent with those obtained by RNA-seq analysis except for miR-423, which was up-regulated in MDSC-D3 compared with MDSC-D1 in RT-qPCR, while it was down-regulated in RNA-seq (Fig. 4). These results indicated that the HiSeq deep sequencing in our study was of high reliability.

### Target prediction for miRNAs

The function of a miRNA is ultimately defined by the genes it targets and by its effect on the expression of these genes. To identify the potential targets of miRNAs, we searched a bovine mRNA (ftp://hgdownload.cse.ucsc.edu/goldenPath/bosTau7/database/refGene.txt.gz) database. Table 5 lists the target genes obtained from MDSC-P, MDSC-D1, and MDSC-D3, which might be regulated by the miRNAs in MDSC-D1, MDSC-D3 and MDSC-P, shown in Additional file 12. The binding energy of the conserved miRNAs with their targets varied from -11.2 to -44.3 kcal/mol and from 10.3 to -49.4 kcal/mol between the novel miRNAs and their targets (Additional file 13). Some miRNAs had more than 1000 predicted targets, and some other target genes were putatively regulated by more than two miRNAs.

**Table 5:** Summary of known miRNA and novel miRNA target prediction

	sample	miRNA number	Target number
known miRNA	MDSC-P	439	962,279
	MDSC-D1	394	857,309
	MDSC-D3	392	861,755
novel miRNA	MDSC-P	42	87,921
	MDSC-D1	17	41,798
	MDSC-D3	20	43,423

### GO enrichment analysis and KEGG pathway analysis of target genes

**Fig. 5:**
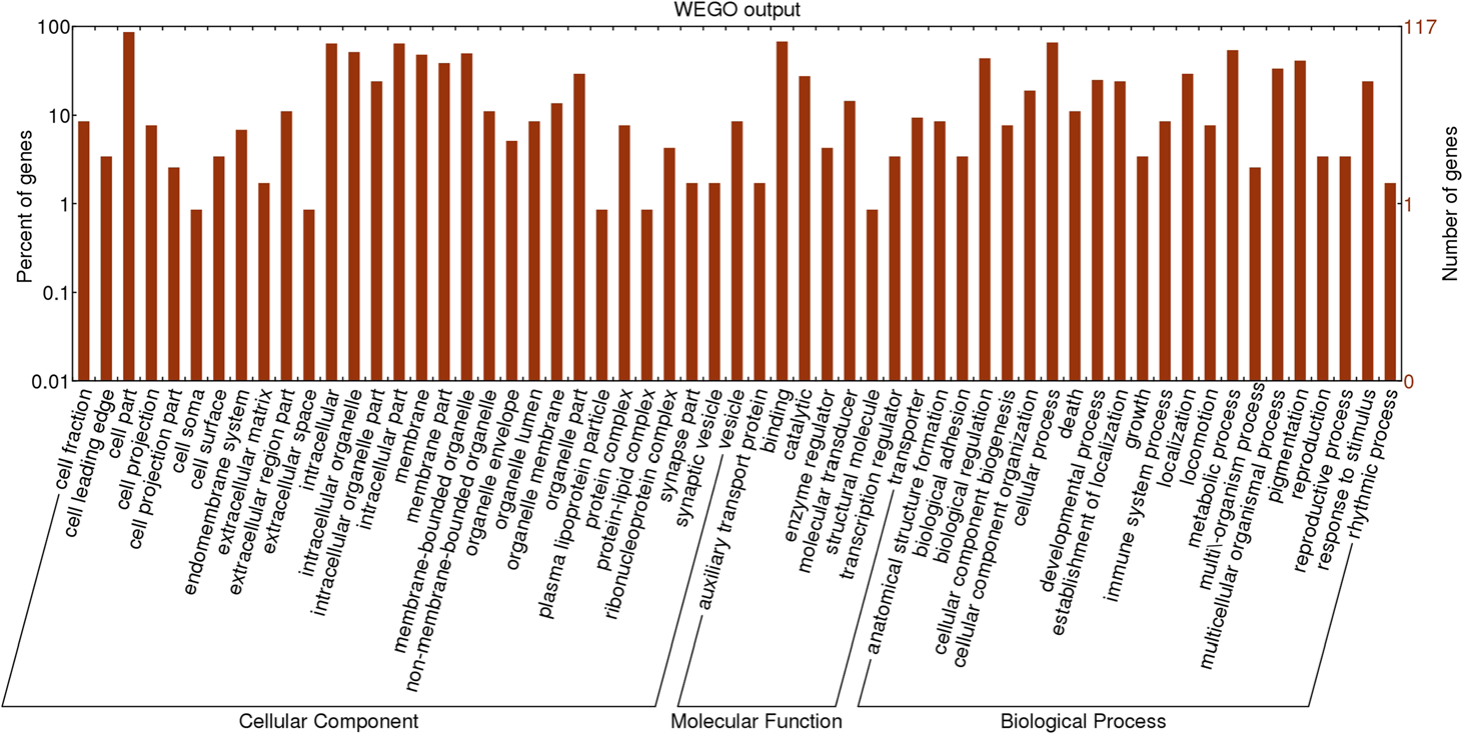
GO analysis based on miRNA-targeted genes. The GOs targeted by abundant miRNAs were shown. The horizontal axis is the GO category and the vertical axis is number of genes

To understand the biological function of miRNAs in MDSCs, all the predicted target genes were classified according to KEGG functional annotations, which help to identify pathways that were actively regulated by miRNAs in MDSCs. Most of these genes were involved in cellular metabolism, pathways in cancer, actin cytoskeleton regulation and the MAPK signaling pathway (Table 6). The most commonly indicated pathway was the metabolic pathway, with 1321 genes representing 12.44 % of the total, followed by the pathways in cancer (3.51 %), biosynthesis of secondary metabolites (3.46 %), regulation of actin cytoskeleton (3.17 %) and the MAPK signaling pathway (3.03 %). All the predicted target genes were submitted for Gene Ontology (GO) analysis using an online version of the Blast2GO program (www.Blast2GO.com). In total, 10,887 genes were termed good or better than 1 using the component ontology with P value analysis, 10,228 genes were assigned different functions and 10,039 genes were termed on biological processes (Additional file 4: Table S2). To determine the potential functions of the different expression miRNAs, the 45 most abundant miRNAs in the three libraries were selected for Gene Ontology analysis. The main GO categories targeted by different expression genes included developmental process, cell death, growth, reproductive process, and metabolic process (Fig. 5). Further analysis for miRNA targets is needed and will help us to gain insight into the roles of these miRNAs in MDSC differentiation.

**Table 6:** The 10 most-enriched KEGG pathways for the target genes of known miRNAs

Pathway	Target genes with pathway annotation	*P*-value	Pathway ID
Metabolic pathways	1321 (12.44 %)	0.4507364	ko01100
Pathways in cancer	373 (3.51 %)	0.8069502	ko05200
Biosynthesis of secondary metabolites	367 (3.46 %)	0.8097889	ko01110
Regulation of actin cytoskeleton	337 (3.17 %)	0.8241077	ko04810
MAPK signaling pathway	322 (3.03 %)	0.8313458	ko04010
HTLV-I infection	297 (2.8 %)	0.8435271	ko05166
Endocytosis	292 (2.75 %)	0.8459811	ko04144
Focal adhesion	285 (2.68 %)	0.8494267	ko04510
Tight junction	266 (2.51 %)	0.8588381	ko04530
Tuberculosis	262 (2.47 %)	0.8608305	ko05152
Phagosome	251 (2.36 %)	0.8663294	ko04145
Epstein-Barr virus infection	244 (2.3 %)	0.869844	ko05169
Neuroactive ligand-receptor interaction	239 (2.25 %)	0.8723616	ko04080
Cytokine-cytokine receptor interaction	237 (2.23 %)	0.8733703	ko04060
Influenza A	236 (2.22 %)	0.873875	ko05164

## Discussion

A series of regulatory changes in gene expression occur during skeletal muscle growth and development [3, 5, 23, 24], and decipherment of these changes is essential for the production of high-quality meat products [25]. The miRNAs are a class of gene expression regulatory factors that play critical roles in muscle cell differentiation [26–29], and identifying the differentially expressed miRNAs is an important first step to investigating the function of miRNAs in the course of MDSC differentiation by high throughput sequencing [30]. In this study we used a next-generation sequencing platform to determine the identities and expression levels of different miRNAs involved in muscle development in an in vitro cell culture system. To investigate microRNAs reliably, we collected 9 plates of cells (isolated from hind-limb muscles of 3 newborn Chinese Simmental calves) from each of the MDSCs in different stages of differentiation. Total RNA was extracted and pooled to identify known and novel miRNAs. In total, 617 miRNAs, including 53 novel miRNA candidates, were identified, and 322 miRNAs were common to all three stages, whereas 117 miRNAs only occurred in MDSC-P, 72 miRNAs only in MDSC-D1, and 70 miRNAs only in MDSC-D3 (Additional file 2).

All conserved miRNAs which were identified in the three libraries belonged to 237 miRNA families. The identified miRNA families have been shown to be conserved in a variety of species. For example, the let-7, miR-25, miR-1, miR-10, miR-8, miR-9, and miR-124 families have been found in 68, 68, 69, 70, 71, 72, and 73 species, respectively, while the miR-2484, miR-2300, miR-2319, miR-2329, miR-2363, miR-2404, miR-2450, miR-2887, miR-3432, miR-2604, miR-6526, and miR-6536 families have only been detected in cattle (Additional file 8). The miRNA family analysis might suggest a species-specific expression profile of miRNAs.

The finding that most members of conserved miRNA families were expressed in MDSCs supported the idea that regulatory or functional diversification occurred [31, 32]. Different family members also displayed drastically different expression levels. For example, the abundance of the miR-2284 family varied from 1 read (bta-miR-2284 m, bta-miR-2284n, bta-miR-2285c) to 34,204 reads (bta-miR-2284x) with RNA-seq. This was also the case for some other miRNA families, such as bta-let-7 (1442–1,331,479 reads), bta-miR-10 (24–10,950 reads), bta-miR-29 (1–153,254 reads) and bta-miR-181 (1–4694 reads). The expression levels of some miRNA families were similar, such as miR-1271, in which 12, 5, and 2 reads were detected, respectively. The existence of a dominant member in a miRNA family might suggest that the regulatory role of this family was performed by the dominant member at the developmental time when the samples were collected for RNA extraction. Abundance comparisons of different members of a miRNA family might provide valuable information on the role that miRNAs play in that specific stage of MDSCs.

Recent studies showed that myogenic differentiation was regulated by muscle-specific miRNA. miR-1 and miR-206 were upregulated during satellite cell differentiation and promoted myogenesis [28, 33], whereas miR-133 was involved primarily in the promotion of proliferation [9]. In this study, sequence comparison with known miRNAs identified the muscle-specific miR-206 as the most abundant miRNA across all MDSC samples, which represented more than 37.39 % of all miRNAs in MDSC-D3, and represented 10.15 % abundance in proliferating satellite cells. The constant high-level expression at all stages after early differentiation suggested that the role of miR-206 was to repress functions associated with muscle precursor cells. Combined with the facts that miR-206 was lowly abundant in proliferating cells of mouse C2C12 cells, and was reported to be induced during differentiation [34], we proposed that its presence was associated with the switch from precursor to mature muscle cell. Compared with the high abundance of miR-206 in MDSCs, bovine miR-1 showed moderate abundance, and then increased throughout the differentiation. The expression pattern was similar to the one observed for this miRNA in mouse muscle development [9]. These data suggested that miR-1 plays different roles from miR-206 in muscle differentiation; miR-1 could affect the regulation of genes that require inactivation in later stages, while miR-206 could have a more constant role in repressing genes immediately after differentiation. Interestingly, miR-133 was detected at low levels in MDSC-D1, but higher in MDSC-D3. Based on abundance levels, miR-206 and miR-1 were considered to have a greater role in MDSC differentiation than miR-133 or their targets could be more abundant. In addition, both miR-206 and miR-1 promoted differentiation [9], which suggested that these two miRNAs might have a greater effect than miR-133 in muscle satellite cell differentiation [35].

In addition, the miR-27b expression level was 1.43- and 1.48-fold lower in MDSC-D1 and MDSC-D3, respectively, than in MDSC-P. The expression of miR-27b was consistent with the previous report of reduced myostatin expression through targeting the 3’-UTR, resulting in myoblast proliferation [36]. On the other hand, miR-27b was reported to be highly expressed in mouse satellite cells after differentiation [11]. Thus, the molecular function of miR-27b might be divergent, depending on the type of muscles, as well as animal species.

In addition to muscle-specific miRNAs, a larger number of ubiquitous miRNAs were present in all libraries. Bta-miR-99a-3p, bta-miR-2396, bta-miR-139, and bta-miR-10a presented at 2-fold greater abundance in MDSCs than in MDSC-P (Table 4). These miRNAs were implicated in multiple cellular processes, including inhibition of growth (miR-99a and miR-139), induction of apoptosis (miR-99a and miR-139), cell cycle arrest (miR-99a), and promotion of differentiation (miR-10a) [37–41]. In addition, miR-181a was up-regulated in MDSC-D1, which promoted apoptosis by targeting Bcl-2 [42] and suppressed tumor growth by targeting the MAPK-Snai2 pathway [43]. miR-503 (log_2_ (MDSC-D3/MDSC-P) = 1.39) suppressed cell proliferation and cell cycle progression, and promoted cell cycle quiescence by targeting cyclin D1 [44]. Conversely, 143 miRNAs presented at 2-fold lower abundance in MDSC-D1 than in MDSC-P; for example, miR-184 (log_2_ (MDSC-D3/ MDSC-P) = –2.29) promoted cell proliferation by targeting c-Myc [45]. Nevertheless, the role of these miRNAs during differentiation of MDSCs is yet to be fully determined.

In addition to the 564 known miRNAs, we also discovered 53 novel bovine miRNAs. Sixteen of the novel miRNAs were found to be conserved among mammals, including rat, mouse, human, rhesus monkey, chimpanzee, pig and dog. The 37 novel bovine miRNAs had not been identified in any other species and may be specific to cattle or skeletal muscle. The novel miRNAs of bta-n25 were relatively abundant in MDSC-P and MDSC-D3, and therefore might be involved in MDSC differentiation.

The results of GO term analysis showed further comprehensive biological processes that miRNA regulated in MDSC differentiation. In total, 10,228 genes were assigned different functions and 10,039 genes were classified into biological processes; this might be because the target genes were predicted. The distributions of GO term categories were similar for the target genes of miRNAs in MDSC-P, MDSC-D1 and MDSC-D3. The biological functions identified by the Blast2GO program included categories related to a wide variety of physiological and biological events, such as cellular developmental processes, metabolic processes, biological regulation and cell differentiation. Further analysis is needed about miRNA target genes which are regulated by muscle-specific miRNAs and would help us to gain insight into the roles of these miRNAs in MDSC differentiation.

Skeletal muscle is known to be one of the major tissues accounting for energy expenditure; both proliferating and differentiating cells must rapidly generate new biomass in the form of nucleotides, proteins and phospholipids to support rapid cell division and growth. The miRNAs regulate a variety of physiologic processes, including glucose and lipid metabolism [46, 47]. To date, several miRNAs have been reported to regulate lipid metabolism, including miR-122, miR-33a, miR-133, miR-335, miR-125a, and miR-34a [48]. These miRNAs were down-regulated in MDSC-D1 in our study. miR-33 regulated genes associated with β-oxidation of fatty acids, including HADHB, CROT and CPT1A [49]. Several studies have demonstrated that miR-122 plays a regulatory role in lipid metabolism. Transfection of an anti-sense oligonucleotide inhibitor of mmu-miR-122 into a mouse hepatocyte-derived cell line (AML12) caused an increase of mRNA of six genes, ie GYS1, SLC7A1, MINK1, ALDOA, CCNG1 and P4HA1 [50], some of which might have indirect effects on lipid metabolism. The miR-335 expression level was closely correlated with expression levels of adipocyte differentiation markers such as PPARγ, aP2, and FAS in 3 T3-L1 adipocytes [51]. Moreover, forced expression of miR-133 decreased GLUT4 expression and reduced insulin-mediated glucose uptake in cardiomyocytes [52]. Overexpression of miR-29a-c in primary hepatocytes and mouse livers decreased the protein levels of PGC-1a and G6 Pase [53]. MiR-195-5p decreased T24 cell glucose uptake, inhibited cell growth and promoted cell apoptosis through suppression of GLUT3 expression [54]. Ectopic expression of miR-27 inhibited the expression of PPARγ and C/EBPα in 3 T3-L1 cells [55]. MiRNA expression patterns may differ by tissue type, suggesting that this mechanism of gene expression regulation was dynamic and may be highly specific, indicating the need for further study to determine their importance in MDSC differentiation.

## Conclusions

This study confirmed the authenticity of 564 known miRNAs, and discovered 53 novel miRNAs in MDSCs during differentiation using RNA-seq. This study expanded the repertoire of bovine miRNAs and could initiate further research in bovine muscle differentiation. In addition, our results revealed distinct miRNA expression patterns during the time course of MDSC proliferation and differentiation, cell cycle progression, cell apoptosis and metabolic pathways. These results would warrant further investigation to determine the regulatory roles of the differentially expressed miRNAs identified in the present study in controlling muscle differentiation.

## Abbreviations

3′ UTRs, 3′ untranslated regions; DM, differentiation medium; DMEM, Dulbecco’s modified Eagle’s medium; GM, growth medium; GO, gene ontology; KEGG, Kyoto Encyclopedia of Genes and Genomes; MDSCs, skeletal muscle-derived satellite cells; MFEs, minimal free energies; miRNAs, microRNAs; RT-qPCR, reverse transcription quantitative real-time PCR

## Additional files

Additional file 1: Primers and sequences for RT-qPCR analysis (XLS 12 kb)

Additional file 2: Bovine conserved miRNAs. Note: The data in this supplementary file can be seen in the Excel spreadsheet. (XLS 273 kb)

Additional file 3: The known miRNA expression profiles between two libraries. Note: The data in this supplementary file can be seen in the Excel spreadsheet.

Additional file 4: Table S1. Top 10 most frequently detected known miRNA in MDSCs. Suppl. **Table S2.** The 10 most-enriched GO categories for the target genes of known miRNAs. (DOC 83 kb)

Additional file 5: Figure S1. Hierarchical cluster analysis of differentially expressed miRNAs during MDSC differentiation. Cluster analysis was performed for differentially expressed miRNAs after data adjustment (log transformation, median center, and normalization). The color codes of green, black, and red represent high, average, and low expression levels, respectively. Note: each row in the figure shows one miRNA, and each column shows one sample pair. So each cell shows the differential expression of a miRNA in one sample pair. In cluster analysis miRNAs that have a similar pattern of differential expression in different sample pairs were clustered together. (JPG 2732 kb)

Additional file 6: Figure S2. Nucleotide bias at each position of sRNA tags. *Note:* miRNA nucleotide bias at each position of MDSC-P (A), MDSC-D1 (B) and MDSC-D3 (C), respectively. Note: each color in the figure shows the sRNA tags whose certain position was a certain base. (JPG 1036 kb)

Additional file 7: Summary of base edit in three libraries. Note: The data in this supplementary file can be seen in the Excel spreadsheet.

Additional file 8: Family analyses of known miRNAs. Note: The data in this supplementary file can be seen in the Excel spreadsheet.

Additional file 9: Novel miRNAs identified in this study. Note: The data in this supplementary file can be seen in the Excel spreadsheet.

Additional file 10: The novel miRNA expression profiles between two libraries. Note: The data in this supplementary file can be seen in the Excel spreadsheet.

Additional file 11: Figure S3. The differential expression of bovine novel miRNAs during different differentiation stages is shown. Compare the novel miRNA expression between different differentiation stages to find out the differentially expressed miRNA. Each point in the figure represents a miRNA. The X axis and Y axis show the expression level of miRNAs in two libraries. Green points represent miRNAs with ratio>2; blue points represent miRNAs with 1/2<ratio≤2; red points represent miRNAs with ratio≤1/2. Ratio=Normalized expression in treatment/Normalized expression in control. (JPG 401 kb)

Additional file 12: Predicted target genes for up-regulation miRNA. Note: The data in this supplementary file can be seen in the Excel spreadsheet.

Additional file 13: Predicted target genes for novel miRNA. Note: The data in this supplementary file can be seen in the Excel spreadsheet.
