# Supplementary material for: Effect of differentiation on microRNA expression in bovine skeletal muscle satellite cells by deep sequencing
Source: Cell Mol Biol Lett. 2016 Jul 28;21:8. doi: 10.1186/s11658-016-0009-x (PMC5415838; doi:10.1186/s11658-016-0009-x)
Supplement: Supplementary file 4 — Top 10 most frequently detected known miRNA in MDSCs. Suppl. Table S2. The 10 most-enriched GO categories for the target genes of known miRNAs. (DOC 83 kb) [file 11658_2016_9_MOESM4_ESM.doc]

Table S1. Top 10 most frequently detected known miRNAs in MDSCs.

| miR-name | MDSC-P  expressed | MDSC-D1  expressed | MDSC-D3  expressed | log_2_ (D1/ P) | log_2_ (D3/ P) | Pre-miRNA | Genomic position | Overlapping transcripts | Clustered miRNA<10kb |
| --- | --- | --- | --- | --- | --- | --- | --- | --- | --- |
| bta-miR-206 | 493002 | 974847 | 1890636 | 0.971 | 1.923 | bta-miR-206 | chr23: 24308042-24308127 [+] | intergenic | bta-mir-133b |
| bta-let-7a-5p | 1331479 | 1273469 | 765333 | -0.077 | -0.815 | bta-let-7a-1 | chr8: 86884872-86884951 [+] | sense ENSBTAT00000042360; bta-let-7a-1-201; exon 1 | bta-let-7f-1,  bta-let-7d |
|  |  |  |  |  |  | bta-let-7a-2 | chr15: 33347569-33347640 [-] | sense  ENSBTAT00000042260; bta-let-7a-2-201; exon 1 | bta-mir-100 |
|  |  |  |  |  |  | bta-let-7a-3 | chr5: 117119385-117119458 [+] | intergenic | bta-mir-2443,  bat-let-7b,  bta-mir-3596 |
| bta-let-7f | 995558 | 727085 | 602217 | -0.466 | -0.741 | bta-let-7f-2 | chrX: 96383532-96383614 [-] | sense  ENSBTAT00000008060; BT.101023-201; intron 60 | bta-mir-98 |
|  |  |  |  |  |  | bta-let-7f-1 | chr8: 86885225-86885311 [+] | intergenic | bta-let-7a-1,  bta-let-7d |
| bta-let-7b | 627767 | 808098 | 550558 | 0.352 | -0.206 | bta-let-7b | chr5: 117120185-117120265 [+] | antisense ENSBTAT00000050917; bta-mir-3596-201; exon 1 | bta-let-7a-3, bta-mir-2443, bta-mir-3596 |
| bta-miR-1 | 69315 | 87798 | 408668 | 0.329 | 2.544 | bta-miR-1-1 | chr13: 55237544-55237619 [-] | intergenic |  |
|  |  |  |  |  |  | bta-miR-1-2 | chr24: 34841096-34841180 [+] | antisense  ENSBTAT00000009822; MIB1-201; intron 12 |  |
| bta-let-7i | 180815 | 121574 | 108217 | -0.586 | -0.756 | bta-let-7i | chr5: 51209081-51209164 [-] | sense  ENSBTAT00000042373; bta-let-7i-201; exon 1 |  |
| bta-let-7c | 56528 | 105316 | 84253 | 0.885 | 0.559 | bta-let-7c | chr1: 19930459-19930542 [-] | sense  ENSBTAT00000042291; bta-let-7c-201; exon 1 | bta-mir-99a |
| bta-miR-21-5p | 149587 | 92391 | 82370 | -0.708 | -0.877 | bta-miR-21 | chr19: 11033072-11033143 [+] | sense  ENSBTAT00000042276; bta-mir-21-201; exon 1 |  |
| bta-miR-199a-3p | 96205 | 77923 | 79482 | -0.317 | -0.291 | bta-miR-199a-1 | chr16: 40491602-40491705 [-] | intergenic | bta-mir-3120, bta-mir-214 |
|  |  |  |  |  |  | bta-miR-199a-2 | chr7: 16508926-16508996 [-] | sense  ENSBTAT00000042269; bta-mir-3604-1-201; exon 1; antisense  ENSBTAT00000036123; BT.18482-201; intron 13 | bta-mir-3604-1 |
| bta-miR-320a | 58698 | 79162 | 52110 | 0.419 | -0.188 | bta-miR-320a-2 | chr20: 15213924-15214005 [+] | antisense  ENSBTAT00000061221; RNF180-201; intron 4 |  |
|  |  |  |  |  |  | bta-miR-320a-1 | chr8: 70060384-70060465 [-] | intergenic |  |

Notes: overlapping transcripts: protein-coding and noncoding genes with respect to the Ensembl database; clustered miRNA<10 Kb: clustered microRNA genes within 10 kb distance in *Bos taurus* genome analyzed by miRBase.

Table S2. The 10 most enriched GO categories for the target genes of known miRNAs

|  | Go term for P | P-value | Go term for F | P-value | Go term for C | P-value |
| --- | --- | --- | --- | --- | --- | --- |
| MDSC-P | multicellular organismal process | 1 | protein binding | 1 | intracellular organelle part | 1 |
|  | localization | 1 | ion binding | 1 | macromolecular complex | 1 |
|  | developmental process | 1 | cation binding | 1 | nucleus | 1 |
|  | establishment of localization | 1 | metal ion binding | 1 | nuclear part | 1 |
|  | anatomical structure development | 1 | transferase activity | 1 | organelle part | 1 |
|  | transport | 1 | transition metal ion binding | 1 | membrane-enclosed lumen | 1 |
|  | cellular component organization or biogenesis | 1 | transporter activity | 1 | protein complex | 1 |
|  | regulation of metabolic process | 1 | transferase activity, transferring phosphorus-containing groups | 1 | organelle lumen | 1 |
|  | multicellular organismal development | 1 | substrate-specific transporter activity | 1 | intracellular organelle lumen | 1 |
|  | cellular component organization | 1 | receptor binding | 1 | nuclear lumen | 1 |
| MDSC-D1 | macromolecule modification | 1 | adenyl nucleotide binding | 0.00587 | intrinsic to membrane | 0.03201 |
|  | protein modification process | 1 | adenyl ribonucleotide binding | 0.00745 | membrane | 0.11789 |
|  | cellular developmental process | 1 | small molecule binding | 0.03425 | membrane part | 0.15438 |
|  | cell differentiation | 1 | transferase activity, transferring phosphorus-containing groups | 0.05781 | integral to membrane | 0.27461 |
|  | biological regulation | 1 | nucleotide binding | 0.08383 | cell part | 1 |
|  | regulation of biological process | 1 | purine nucleotide binding | 0.14885 | membrane-bound organelle | 1 |
|  | cellular macromolecule metabolic process | 1 | ribonucleotide binding | 0.18399 | intracellular part | 1 |
|  | regulation of metabolic process | 1 | purine ribonucleotide binding | 0.18399 | organelle | 1 |
|  | cellular metabolic process | 1 | kinase activity | 0.37285 | cytoplasm | 1 |
|  | signaling | 1 | GTPase regulator activity | 0.51546 | nucleus | 1 |
| MDSC-D3 | multicellular organismal process | 1 | small molecule binding | 0.41272 | intrinsic to membrane | 1 |
|  | localization | 1 | adenyl ribonucleotide binding | 0.58673 | organelle lumen | 1 |
|  | developmental process | 1 | adenyl nucleotide binding | 0.76099 | nucleus | 1 |
|  | establishment of localization | 1 | nucleotide binding | 1 | cell part | 1 |
|  | anatomical structure development | 1 | ribonucleotide binding | 1 | membrane part | 1 |
|  | transport | 1 | purine ribonucleotide binding | 1 | membrane | 1 |
|  | cellular component organization or biogenesis | 1 | purine nucleotide binding | 1 | intracellular part | 1 |
|  | regulation of metabolic process | 1 | GTPase regulator activity | 1 | membrane-bound organelle | 1 |
|  | multicellular organismal development | 1 | nucleoside-triphosphatase regulator activity | 1 | organelle | 1 |
|  | cellular component organization | 1 | transferase activity | 1 | cytoplasm | 1 |
