# Supplementary figures and images for: Effect of differentiation on microRNA expression in bovine skeletal muscle satellite cells by deep sequencing
Source: Cell Mol Biol Lett. 2016 Jul 28;21:8. doi: 10.1186/s11658-016-0009-x (PMC5415838; doi:10.1186/s11658-016-0009-x)

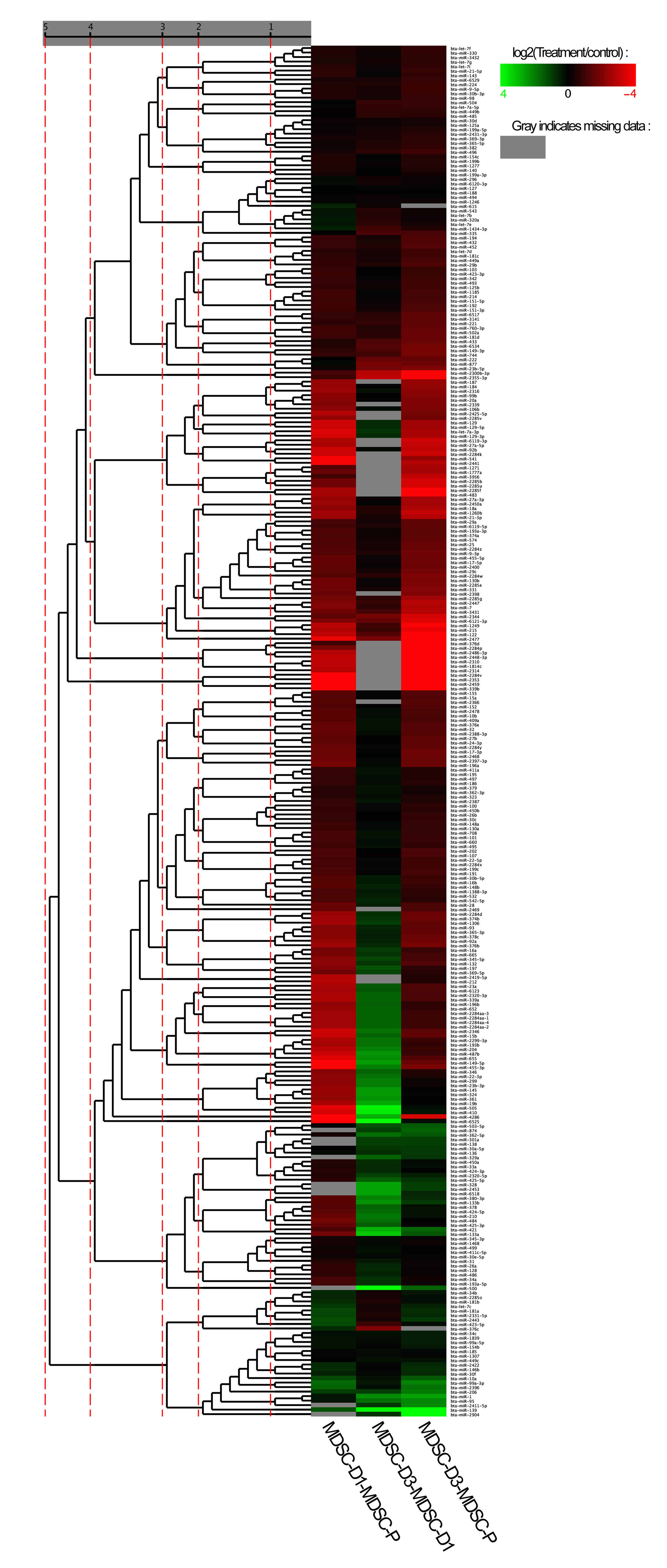

Supplement: Supplementary file 5 — Hierarchical cluster analysis of differentially expressed miRNAs during MDSC differentiation. Cluster analysis was performed for differentially expressed miRNAs after data adjustment (log transformation, median center, and normalization). The color codes of green, black, and red represent high, average, and low expression levels, respectively. Note: each row in the figure shows one miRNA, and each column shows one sample pair. So each cell shows the differential expression of a miRNA in one sample pair. In cluster analysis miRNAs that have a similar pattern of differential expression in different sample pairs were clustered together. (JPG 2732 kb) [file 11658_2016_9_MOESM5_ESM.jpg]

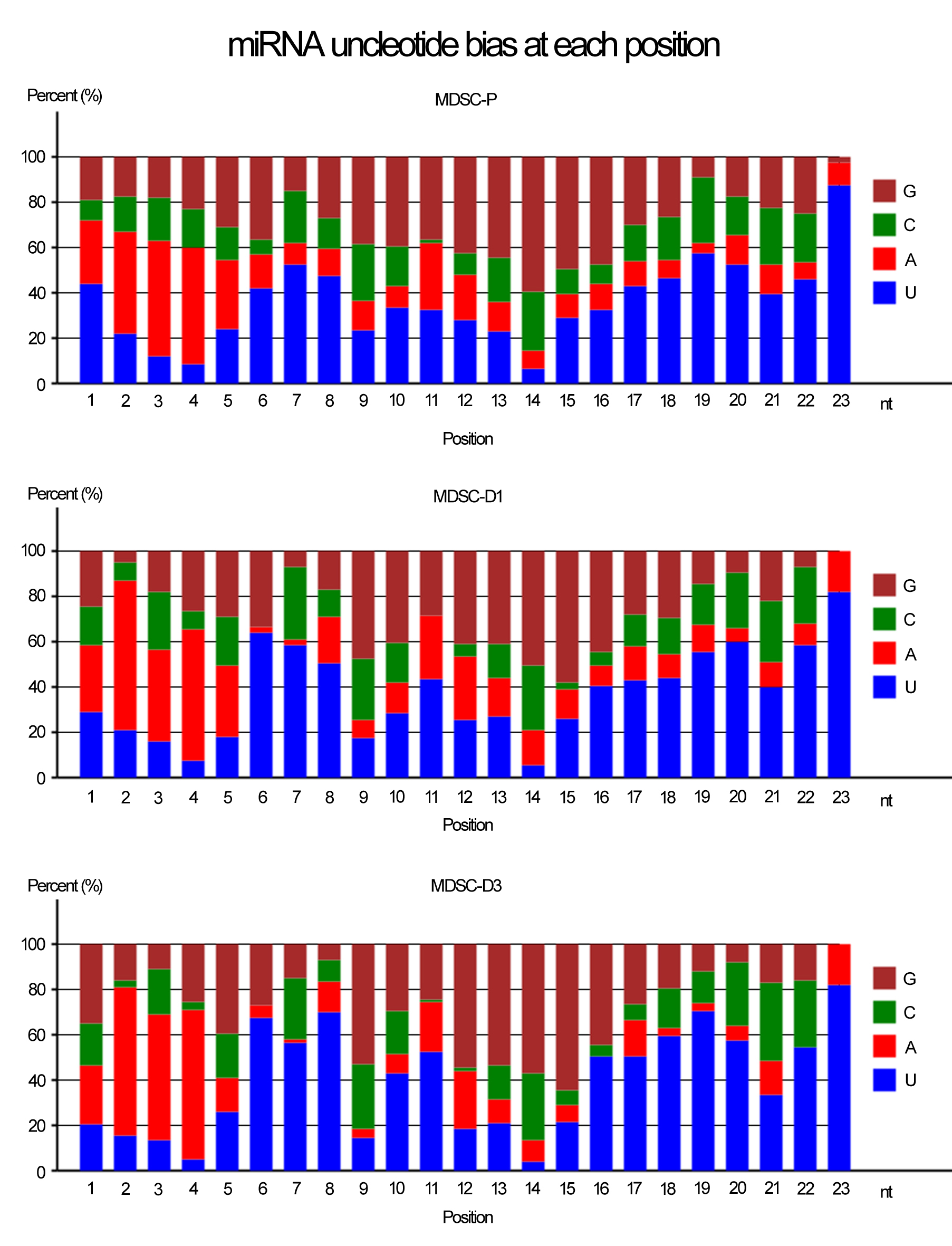

Supplement: Supplementary file 6 — Nucleotide bias at each position of sRNA tags. Note: miRNA nucleotide bias at each position of MDSC-P (A), MDSC-D1 (B) and MDSC-D3 (C), respectively. Note: each color in the figure shows the sRNA tags whose certain position was a certain base. (JPG 1036 kb) [file 11658_2016_9_MOESM6_ESM.jpg]

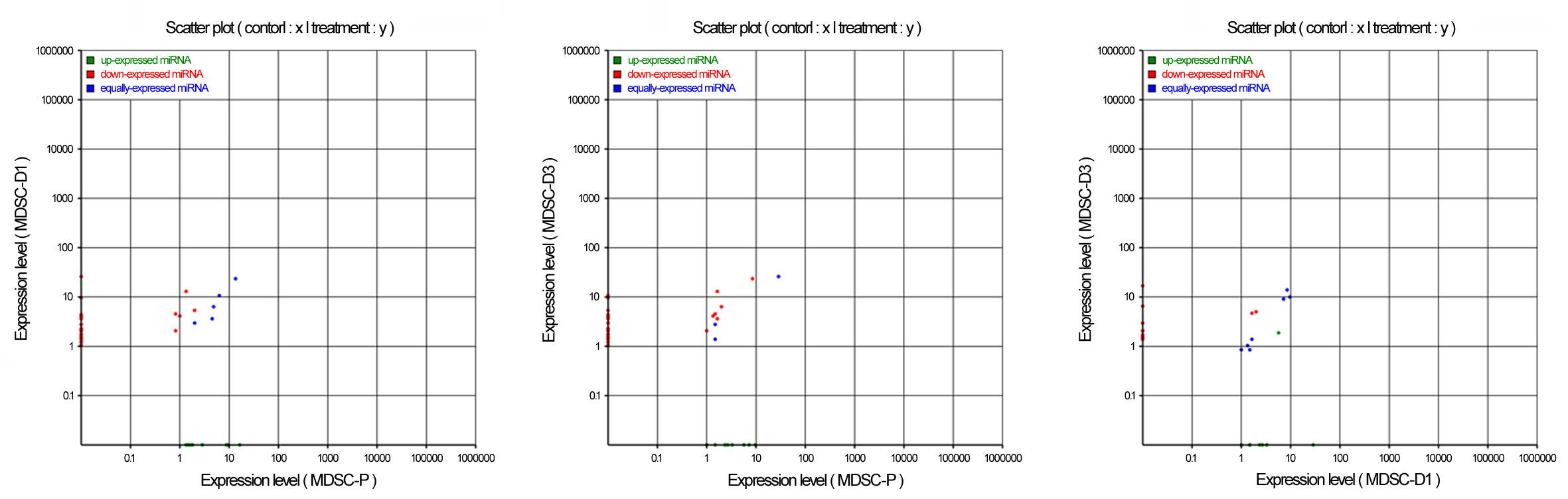

Supplement: Supplementary file 11 — The differential expression of bovine novel miRNAs during different differentiation stages is shown. Compare the novel miRNA expression between different differentiation stages to find out the differentially expressed miRNA. Each point in the figure represents a miRNA. The X axis and Y axis show the expression level of miRNAs in two libraries. Green points represent miRNAs with ratio>2; blue points represent miRNAs with 1/2<ratio≤2; red points represent miRNAs with ratio≤1/2. Ratio=Normalized expression in treatment/Normalized expression in control. (JPG 401 kb) [file 11658_2016_9_MOESM11_ESM.jpg]
